# Acute ischemic stroke triggers a cellular senescence-associated secretory phenotype

**DOI:** 10.1038/s41598-021-95344-5

**Published:** 2021-08-03

**Authors:** Coral Torres-Querol, Pascual Torres, Noemí Vidal, Manel Portero-Otín, Gloria Arque, Francisco Purroy

**Affiliations:** 1 Clinical Neurosciences Group, Institut de Recerca Biomèdica de Lleida (IRBLleida), Lleida, Spain; 2 Experimental Medicine Department, Universitat de Lleida, Institut de Recerca Biomèdica de Lleida (IRBLleida), Lleida, Spain; 3 Department of Pathology, Hospital Universitari de Bellvitge, L’Hospitalet de Llobregat, Barcelona, Spain; 4 Stroke Unit, Department of Neurology, Hospital Universitari Arnau de Vilanova, Medicine Department, Clinical Neurosciences Group IRBLleida, Universitat de Lleida, Avda Rovira Roure 80, 25198 Lleida, Spain; 5 Medicine Department, Universitat de Lleida, Institut de Recerca Biomèdica de Lleida (IRBLleida), Lleida, Spain

**Keywords:** Neuroscience, Stroke

## Abstract

Senescent cells are capable of expressing a myriad of inflammatory cytokines and this pro-inflammatory phenomenon is known as senescence-associated secretory phenotype (SASP). The contribution of this phenomenon in brain ischemia was scarce. A mouse model of transient focal cerebral ischemia by compressing the distal middle cerebral artery (tMCAo) for 60 min was used. SASP, pro-inflammatory cytokines and cell cycle mRNAs levels were quantified at 30-min and 72 h post-surgery. Immunohistochemistry in paraffin embedded human brain slides and mouse brain tissue was performed. Our results showed an increase of both *p16* and *p21* mRNA restricted to the infarct area in the tMCAo brain. Moreover, there was an induction of *Il6*, *Tnfa*, *Cxc11,* and its receptor *Cxcr2* mRNA pro-inflammatory cytokines with a high positive correlation with *p16*/*p21* mRNA levels. The p16 was mainly shown in cytoplasm of neurons and cytoplasm/membrane of microglial cells. The p21 was observed in membrane of neurons and also it showed a mixed cytoplasmic and membranous pattern in the microglial cells. In a human stroke patient, an increase of P16 in the perimeter of the MCA infarct area was observed. These suggest a role of SASP in tMCAo mouse model and in human brain tissue. SASP potentially has a physiological role in acute ischemic stroke and neurological function loss.

## Introduction

An ischemic event is caused by the occlusion within an arterial vessel supplying blood to an area of the brain, resulting in a corresponding loss of neurological function^1^. The local lack of oxygen and nutrients leads to a disarrangement of cellular homeostasis and, eventually, to cell death within the original infarct area. The ischemic microenvironment shows a high vulnerability of neurons and some resistance in glial cells. Although many damaging processes have been described as fundamental on the pathophysiology of an ischemic event, biological aging might also play a role^1,2^. Further, age is associated to a worse prognosis^3,4^.

Cellular senescence is defined as a biological limit in cell replication, but senescent cells can survive longer times. In a late stage, senescent cells are capable of expressing a myriad of NF-κB-regulated inflammatory cytokines^5^, like interleukin 6 (IL-6), IL-8, IL-1ß, TNF-α, interferon-γ, and TGF-β^6^. This condition is known as senescence-associated secretory phenotype (SASP) and it occurs in different cell lines and in neurodegenerative diseases like Alzheimer’s disease^7^, Parkinson’s disease^8^ and amyotrophic lateral sclerosis^9^. SASP factors modulate the surrounding cellular microenvironment by activating the immune system and also by affecting neighboring non-senescent cells promoting malignant transformation via paracrine signaling^10^. Activation of senescence is modulated by two main pathways: the Cdkn2a (p16) and Cdkn1a (p21) signaling cascades, which promote an irreversible cell cycle arrest and cause changes in cell morphology, chromatin organization and in mitochondrial and lysosomal function^11,12^. However, whether SASP factors contribute to ischemic brain injury within the insult area or the cellular phenotype in this biological context is still unknown.

Here, we have investigated the expression of senescence markers in the brain of a preclinical mouse model of ischemic stroke by mRNA gene quantification and immunohistological approach to determine whether cellular stress in stroke is associated with activation of senescence pathways in the brain, specifically determining neuronal and glial expression for cellular senescence-associated proteins, and further describe them in post-mortem brain tissue of ischemic stroke donors.

## Experimental procedures

### Murine stroke model of focal ischemia

Transient middle cerebral artery occlusion (tMCAO) surgical procedure was previously described and detailed surgical procedure is included in the section Extended Methods^13^. Briefly, a glass micropipette was used to compress the MCA for 60 min in CD1 male mice of 12 weeks of age (n = 11). The compression was applied to achieve a steady decrease in regional cerebral blood flow (rCBF) below 75–80% of baseline. After recanalization, animals whose rCBF did not reach to 80% of the baseline were excluded. tMCAO animals were sacrificed 30 min and 72 h after surgery. The animal experiments followed ARRIVE guidelines^14^. Cervical dislocation was the method performed to euthanize mice before extracting the brain for the gene expression analysis.

Mice under anesthetic agent (ketamine/xylazine) were euthanized by exsanguination/cardiac perfusion to obtain perfused brain tissue and perform immunohistochemical characterization.

All procedures conformed to the National Guidelines on Animal Protection and were approved by the Animal Ethics Committee of Universitat de Lleida (CEEA 02-07/17).

### Human samples

Paraffined human brain sections from two ischemic stroke patients coming from the “Banco de Cerebros” of biobank HUB-ICO-IDIBELL (registration number: B.0000609) were analyzed. The biobank HUB-ICO-IDIBELL follows the Comite de Ética de la Investigación (CEIC) del Hospital Univesitari de Bellvitge (Barcelona). Written informed consent was obtained before autopsy. All procedures followed the guidelines of the Declaration of Helsinki developed by the World Medical Association (WMA) regarding ethical principles for medical research involving human subjects. Patient #1 was 79 years old male with a left middle cerebral artery infarct and mortality was due to cirrhosis decompensation. Patient #2 was 47 years old male with a lacunar right Globus pallidus infarct and mortality was due to sepsis. Both patients had a stroke more than 15 days before death.

### Gene expression of SASP and cell cycle markers

Brain samples from tMCAO mice (n = 3 for 30 min; n = 5 for 72 h) were dissected on core, ipsilateral (the area adjacent to the core) and contralateral area. On those three areas, RNA was isolated according to detailed protocol described on Extended Methods. Then cDNA was synthesized with random hexamer primers using the High-Capacity cDNA Reverse Transcription kit (Applied Biosystems, Thermo Fisher Scientific, Spain). Principal transcriptional components of senescence (*p16* and *p21*), SASP (*Il6, Cxcl1, Tnfα, Tgfb2, Cxcr2*) and cell cyle (*Cdk4*) were analyzed. Real time quantitative polymerase chain reaction (RT-qPCR) experiments were performed using a CFX96 instrument (Bio-Rad, Hercules, California, USA) with SYBR Master Mix (Thermo Fisher Scientific, #4472908). Primers sequences are included in Table S1. All samples were run in duplicates. 2^ΔΔ^Ct method was used to quantify mRNA levels of expression and fold changes were calculated relative to *Actb* gene as an endogenous reference gene.

### Immunohistochemical characterization of p16 and p21

Animals (n = 3) were transcardially perfused with 4% paraformaldehyde (PFA) 72 h after surgery and brain frozen coronal sections obtained (16 μm). Immunohistochemistry was performed against mouse anti-p16 (1:200; Abcam, Cat. No. ab54210), mouse anti-p21 (1:200; Santa Cruz Biotechnology, Cat. No. sc-817), rabbit anti-GFAP (1:200; Abcam, Cat. No. ab7260), rabbit anti-Iba1 (1:200; FUJIFILM Wako, Cat. No. 019-19741), rabbit anti-NeuN (1:200; Abcam, Cat.No.ab177487), rabbit anti-NF-κB p65 (1:200; Proteintech, Cat. No. 10745-1-AP), chicken anti-Iba1 (1:200; Synaptic Systems, Cat No. 234 006), and rabbit gamma H2AX [p Ser139] antibody (EP854(2)Y, 1:200; NOVUS Biologicals, Cat. No. NB100-79967) primary antibodies and followed by appropriated secondary antibodies. Samples were captured using a laser scanning confocal microscope (Olympus FV1000). Adequate controls (secondary antibody only) gave no specific signals. Images were analyzed in a double-blinded fashion. NF-κB colocalization with Iba1-positive cells and cellular expression of individual p16 and p21-positive cells were determined with Fiji software^15^. Quantification of protein intensity was performed by CellProfiler software^16^.

### Statistical analysis

Data were analyzed using the Statistical Package for the Social Sciences (version 21.0, SPSS Inc.). mRNA expression studies (cellular senescence and cell cycle markers) and protein expression intensity were defined by either one‐way ANOVA or paired Student's t‐test as appropriate. Data are expressed as means ± s.e.m. A *p* value of < 0.05 was considered statistically significant. Data was plotted using GraphPad Prism 5.04 (GraphPad Software, Inc., La Jolla, CA, USA) and MetaboAnalyst^17^.

### Ethics approval

Human tissue was obtained from biobank HUB-ICO-IDIBELL (registration number: B.0000609). All procedures performed in studies involving animals were in accordance with the ethical standards of the animal Ethics Committee of Universitat de Lleida (CEEA-020220).

## Results

### mRNA expression of SASP and cell cycle markers in tMCAO mouse model

**Figure 1:**
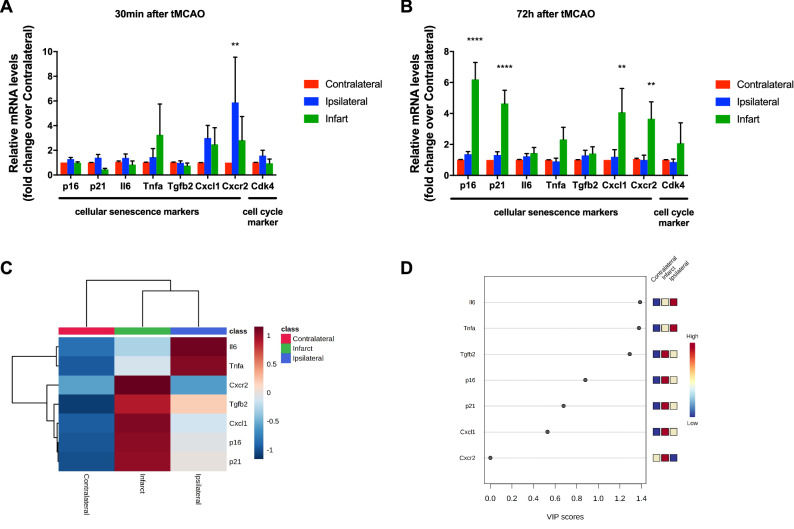
mRNA levels of a set of SASP and inflammatory markers. mRNA levels of a set of cellular senescence markers (fold change over contralateral) are increased and correlated in infarct core at 72 h after tMCAO. (**A**) qRT-PCR analyses of *p16*, *p21*, *Il6*, *Tnfa*, *Tgfb2, Cxcl1, Cxcr2* and *Cdk4* comparing infarct, ipsilateral and contralateral regions of MCAO mouse model at 30 min after tMCAO. (**B**) qRT-PCR analyses of *p16*, *p21*, *Il6*, *Tnfa*, *Tgfb2, Cxcl1, Cxcr2* and *Cdk4* comparing infarct, ipsilateral and contralateral regions of MCAO mouse model at 72 h after tMCAO. (**C**) Heatmap of analyzed regions, average sample expression. (**D**) Variable importance in projection (VIP) plot.

At 30 min post-surgery, there was an increase of *p16*, *p21*, *Il6*, *tnfa*, *Cxcl1* and *Cdk4* on the ipsilateral region compared to the contralateral but no significant differences were found. Only the chemokine receptor *Cxcr2* was significantly increased on the ipsilateral region. The expression of *Tnfa* mRNA was higher on the infarct area, nor differences did not reach statistical significance (Fig. 1A).

The *p16*, *p21*, *Cxcl1* and *Cxcr2* mRNA expression increased significantly on the infarct area of the cerebrum at 72 h after injury, whereas the other transcriptional components were not differently expressed on the analyzed regions (6.09-fold increase for p*16*, 4.63-fold increase for *p21*, 4.07-fold increase for *Cxcl1* and 3.65-fold increase for *Cxcr2* over contralateral; *p* < 0.05) (Fig. 1B). The *Il6*, *Tnfa, Tgfb2* and *Cdk4*, showed a trend to increase in the infarct area but differences did not reach statistical significance. Interestingly, the contralateral area showed a similar expression pattern for all genes and the ipsilateral and infarct areas showed a clear clustering expression. The *Il6* and *Tnfa* correlate positively on the ipsilateral area whereas *p16*, *p21*, *Cxcl*, *Cxcr2 and Tgfb2* correlate positively on the infarct area. The expression profile of these components clustered the different regions as it was shown in the heatmap at 72 h after tMCAO (Fig. 1C). VIP score analysis (> 1) identified *Il6*, *Tnfa* and *Tgfb2* genes that most contributed to the variation of the model at 72 h after tMCAO (Fig. 1D).

### p16, p21, p65 NF-κB and γH2AX cellular distribution in tMCAO mouse model

**Figure 2:**
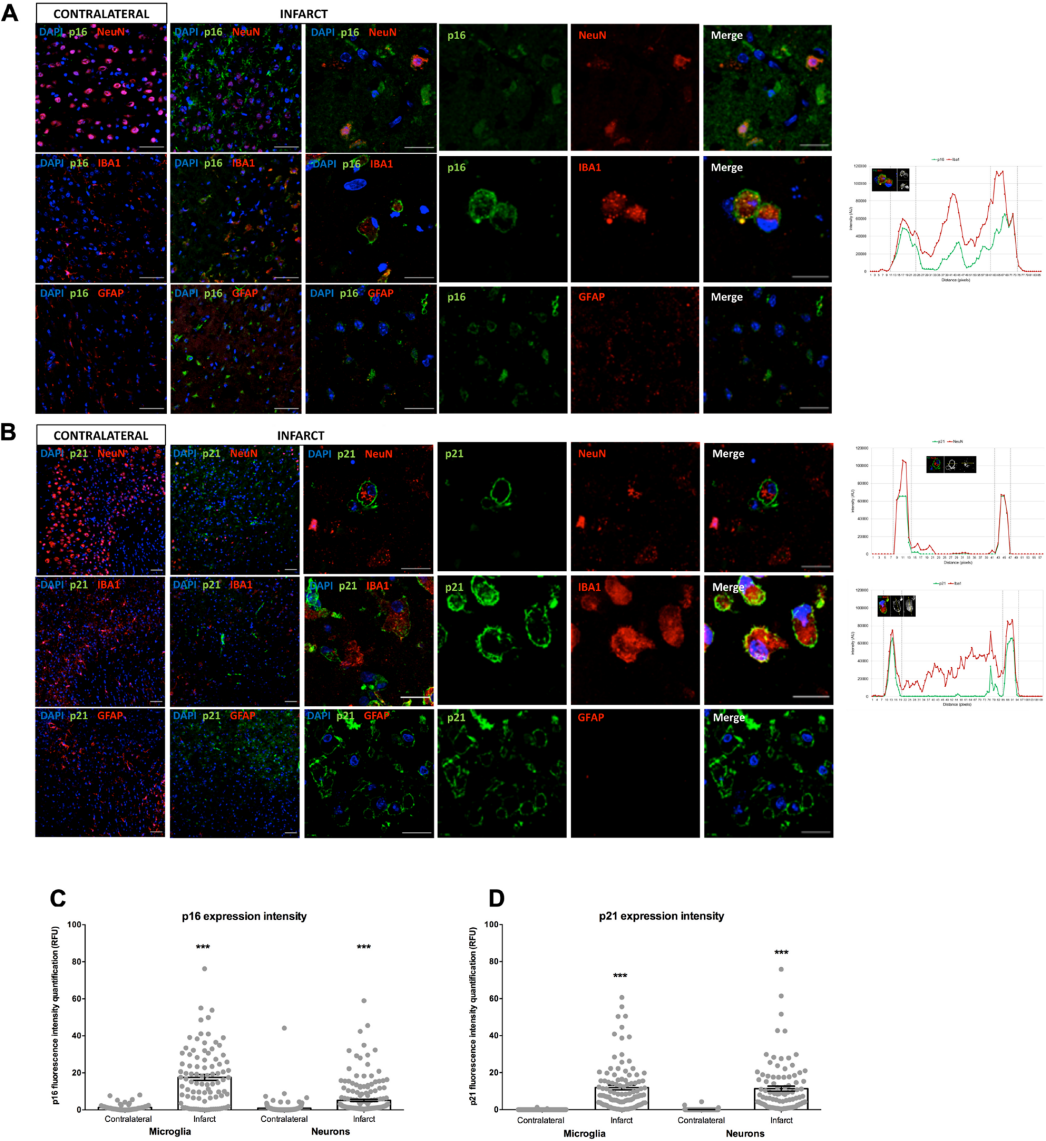
*p16* and *p21* subcellular location in a preclinical animal model of stroke. (**A**) Double immunofluorescence for co-localization of *p16* (green), NeuN/IBA1/GFAP (red) and DAPI (blue). Intensity expression plot showed the cellular distribution of p16 in microglia. (**B**) Double immunofluorescence for co-localization of *p21* (green), NeuN/IBA1/GFAP (red) and DAPI (blue). *p16* and *p21* immunostained cells could be observed in the infarct area at 3 days after tMCAO. Intensity expression plot showed the cellular distribution of p21 in microglia and neurons. (**C**) p16 expression intensity in microglia and neurons on contralateral and infarct areas. (**D**) p21 expression intensity in microglia and neurons on contralateral and infarct areas. Scale bar contralateral and infarct = 50 µm. Scale bar infarct zoom = 20 µm.

To determine whether senescence markers (*p16* and *p21*) are expressed in specific cell types, antibodies recognizing neurons (NeuN marker), microglia (Iba1 marker) and astroglia (GFAP marker) were used. The p16 and p21 positive cells were found in the infarct area among neurons and microglia at 72 h after tMCAO injury. However, p16 and p21 were not expressed in the contralateral area of the brain. Strikingly, the expression of p16 and p21 positive cells in neurons and microglia showed a cytoplasmic and membranous pattern (Fig. 2A,B and S1).

The subcellular intensity localization of p16 and p21 was analyzed in individual neurons and microglia. A higher p16 and p21 expression in the infarct area was observed, and p16 showed a granular cytoplasmatic expression in microglia and neurons, while p21 showed a membrane expression in neurons. The total expression intensity of p16 (Fig. 2C) and p21 (Fig. 2D) in those cells was higher in the infarct area (p16 microglia: *p* < 0.0001; p16 neurons: *p* < 0.0001; p21 microglia: *p* < 0.0001; p21 neurons: *p* < 0.0001).

**Figure 3:**
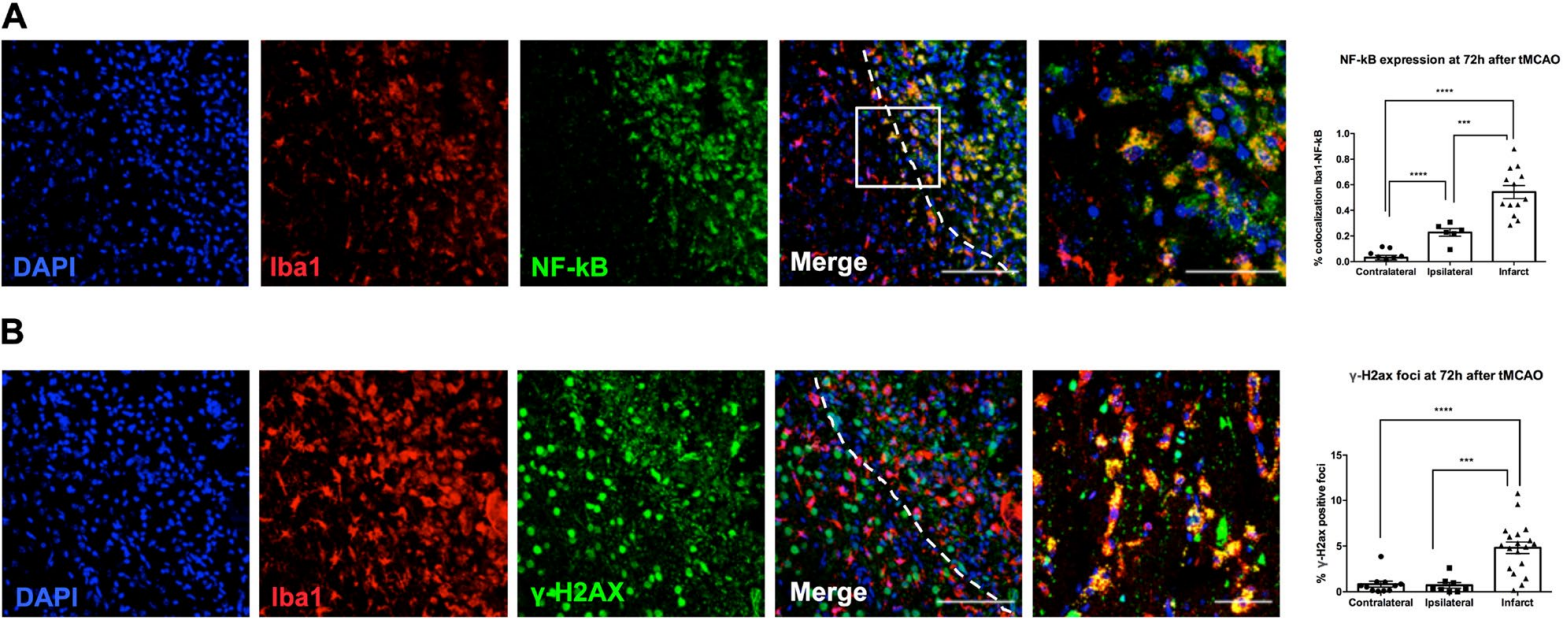
Microglial cells of the ischemic area showed a higher expression of NF-kB and γ-H2AX. (**A**) Immunofluorescence NF-kB expression showed an infarct area location, delimited by white dotted line. Plot represented the NF-kB expression by areas (contralateral vs infarct area: *p* < 0.0001; ipsilateral vs infarct: *p* = 0.0009; contralateral vs ipsilateral: *p* < 0.0001). Scale bar = 100 µm; insert scale bar = 40 µm. (**B**) Nuclear expression of γ-H2AX in microglial cells. Plot represented the γ-H2AX positive foci in contralateral, ipsilateral and infarct areas (contralateral vs infarct: *p* < 0.0001; ipsilateral vs infarct: *p* = 0.0004). Scale bar = 100 µm; insert scale bar = 50 µm.

To determine the activation of an important pro-inflammatory pathway, related to SASP and neuroinflammation, we analyzed the expression of p65 NF-κB in microglia. An increase in the correlation coefficient (Rcoloc) of this marker and Iba1 (microglia) was observed in infarct and ipsilateral area (Fig. 3A). To analyze upstream pathways elements of stress-induced senescence, we determined the expression of DNA reparation foci (γH2AX marker) in microglia. This marker was significantly increased in infarct area and remains unchanged between contralateral and ipsilateral area (Fig. 3B).

### Senescence-associated-β-galactosidase (SA-β-gal) staining in tMCAO mouse model

SA-β-gal activity, a common marker of cellular senescence, was used in tMCAO mice tissue brains to identify senescent cells at 72 h after surgery. No SA-β-gal positive cells were observed in the infarct area nor in the contralateral area of each brain (Fig. S2).

### P16 and P21 immunohistochemistry in post-mortem human stroke samples

**Figure 4:**
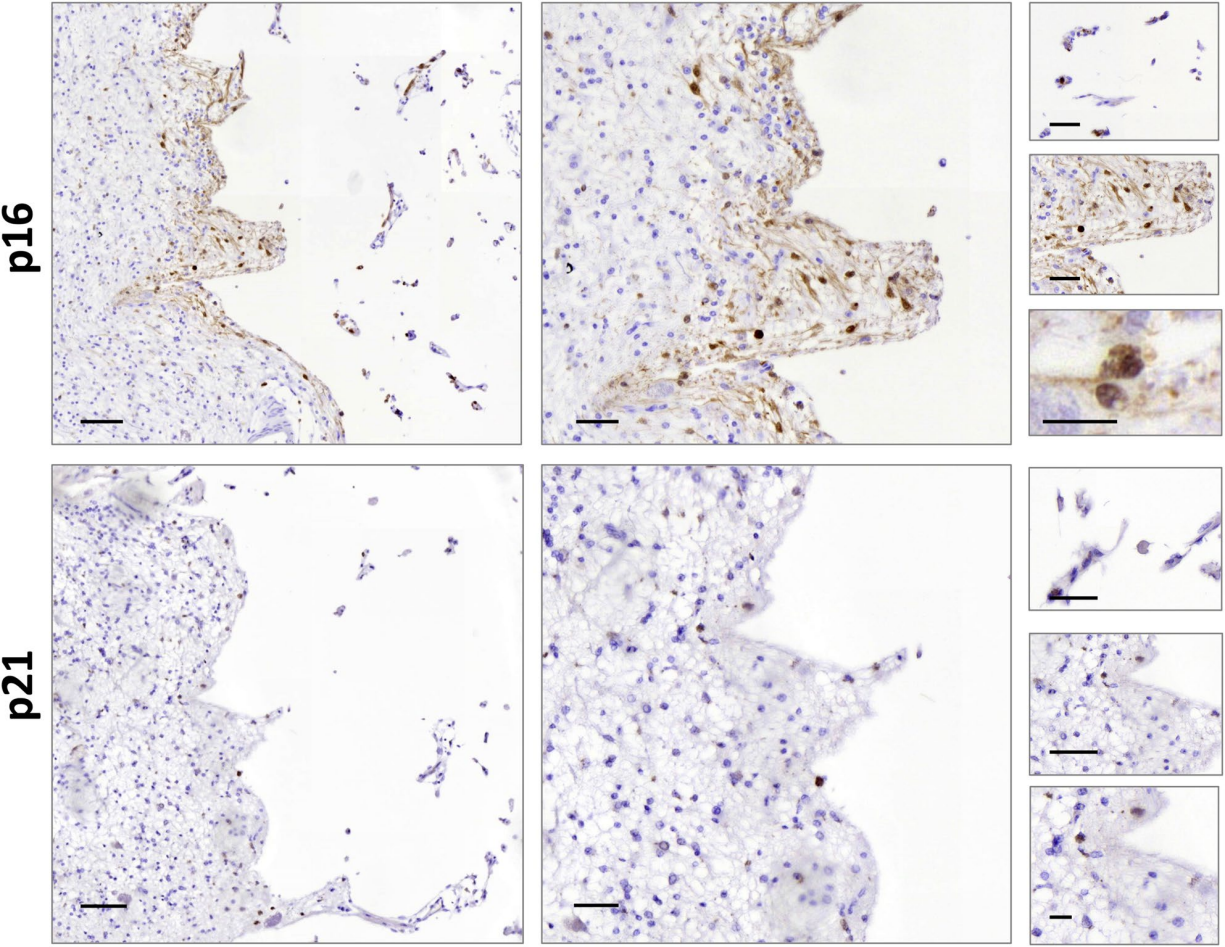
P16 and P21 subcellular location in MCA ischemic stroke patient’s brain tissue. Scale bar left images = 100 µm, middle images = 50 µm and right images = 20 µm.

As cellular senescence-associated secretory phenotype was triggered by an ischemic event in a preclinical model, we investigated whether this phenotype would also be observed in human brains. Post-mortem brain samples of stroke donor with a left middle cerebral artery infarct were analyzed. Immunohistochemistry against P16 and P21 was performed and the adjacent area of the ischemic core showed a positive immunoreactivity for P16 and P21, with a nuclear and cytoplasmic expression pattern and extracellular aggregated, respectively. A higher number of P16 positive cells were observed in the perimeter of the infarct area (60.32% P16 positive cells in the perimeter of the infarct area vs 7.79% in the rest of the section) (Fig. 4). Samples of a second stroke donor with globus pallidus infarct were also analyzed, but P16 and P21 immunoreactivity was weaker within the infarct area (Fig. S3).

## Discussion

Recently, a concept which connects cellular senescence and age-related tissue dysfunction has emerged^18^. Indeed, in the central nervous system, involvement of senescence in neurons, astrocytes and microglia is increasingly recognized in the development of brain aging and/or age-related neurological disorders^19^. However, whether cellular senescence impacts ischemic stroke has not been well established. In this study, we have described for the first time the SASP phenomenon in ischemic stroke. We have showed that the gene expression of senescence markers (*p16* and *p21*) and pro-inflammatory cytokines (*Il6, Cxcl1*, *Tnfa* and *Cxcr2*) in the brain of tMCAO mouse is restricted to the dissected infarct area 72 h after the ischemic insult. Moreover, p16 *and* p21 are expressed in neurons and microglial cells within the infarct area with both cytoplasmic and membranous expression pattern in tMCAO mouse brain, in contrast with the expected nuclear distribution. Finally, an increase of P16 in the perimeter of the infarct area was observed in post-mortem human brain tissue of ischemic stroke donor.

Senescence and SASP has been described in several age-related pathologies^20^. However, it has not been described in ischemic stroke. Our results are compatible with SASP transcriptional profile, characterized by an increase of cell cycle inhibitors (*p16* and *p21*) and pro-inflammatory cytokines^21^. Although previous authors have described a neuroinflammatory profile in ischemic stroke, our novelty is the description of stress-induced senescence triggering a neuroinflammatory profile, which is compatible with SASP phenomenon. These changes related to gene synthesis are restricted to ischemic core, where cytokines are synthesized and probably diffused across the tissue in a paracrine manner, and those results are not related with cell cycle roles of the genes. The role of *p16* and *p21* expression in senescence and inflammatory process is controversial. While both *p16* and *p21* are described as the main inducers of senescence^22^ and therefore participating in SASP and chronic inflammation^6^, some authors demonstrate the anti-inflammatory and pro-resolution role of p16 and p21 in a complex interaction with CDK and NF-κB pathway (reviewed in^23^), which is increased concomitantly with p16 and p21 in microglial cells in our mouse model. Moreover, a high DNA reparation focus in infarct is compatible with the upregulation of p21 expression within DNA Damage Response. This regulation is important to maintain the survival of senescent cells under stress conditions^15^. In contrast to canonical senescence associated with aging, which implies a long period of time and the persistence of chronic inflammation, *p16* and *p21* pro-resolution effects are seen in acute stress models. This may reflect an important role of the time regulating the functions of *p16* and *p21* in senescence and inflammation and could have implications in ischemic stroke. In transient forebrain ischemia, elevated p21 might be a response to the ischemic insult to protect the neurons by arresting them in first steps of the cell cycle. The p21 expression is enhanced in surviving neurons surrounding dead area^24^. Protein subcellular location is also relevant for p16 function. The cytoplasmic immunoreactivity of p16 appears to be an unfavorable prognostic indicator in high-grade astrocytoma patients^25^. Moreover, expression of P16 and P21 in glia suggests senescence induction, but neuronal p21 expression might reflect a more general mechanism of age‐related cell cycle dysregulation in the frontal association cortex of amyotrophic lateral sclerosis patients^26^. Several cell cycle independent functions of p16 and p21 have been described. Interestingly, p16 membrane localization regulates cytoskeleton in endothelial cells^27^ and its depletion causes defects in motility, morphogenesis and cytoskeletal organization^28^. Herein, microglia migration and phagocytosis could be regulated by similar mechanism after ischemic insult.

A recent study shows a similar SASP transcriptional profile in trauma brain injury (TBI) mouse model^29^. In contrast with our results, it has observed an increase in SA-β-gal activity which increases at 4, 7- and 14-days post-stroke. This would explain the lack of SA-β-gal activity in our model (3 days after ischemic stroke) due to a shorter post-ischemic time window.

The SASP hypothesis, in which the senescent cells that accumulate in body tissues over time contribute to inflammation progression and functional decay in the elderly has recently been proposed^30^. However, how is it related to stroke progression remains unclear. Ischemic stroke is characterized by the disruption of cerebral blood flow, which produces a central core of dead neurons surrounded by a penumbra of damaged but partially functional neurons. As we have observed, during the first 30 min after ischemic stroke, cellular senescent markers were increased in the penumbra area and over time were higher in the ischemic core. This would be related with stroke progression. If an ischemic event would accelerate the SASP phenomena in a cellular environment, that would be related with the hypoxia as an apoptotic mechanism contributing to the senescence phenotype and it would also be related with further neurodegeneration of alive cells and selective loss of neurological function. We have demonstrated the role of P16 in human MCA stroke but not in lacunar stroke. This could be explained by the notorious differences in the pathology of lacunar and non-lacunar strokes^1^.

As for the limitations of the present work, we are aware that a longer period of time after tMCAO would have shown different phenomena and that the number of analyzed human samples is minimal. We should also consider that age constraints could play a role in this pathway, as the mice evaluated are young. Further, experimental senolytic treatments, such as quercetin and navitoclax, among others, it could have led to functional improvement. The evaluation of its translational impact, and the study of ageing phenomena, it is envisaged in the near future. However, doubts on whether these treatments should be given in a preventive scheme (i.e. anticipating the hypoxic environment) or rather, they should be administered in a more clinically amenable manner (i.e. short after the insult) are present and both experimental designs would be beneficial.

**Figure 5:**
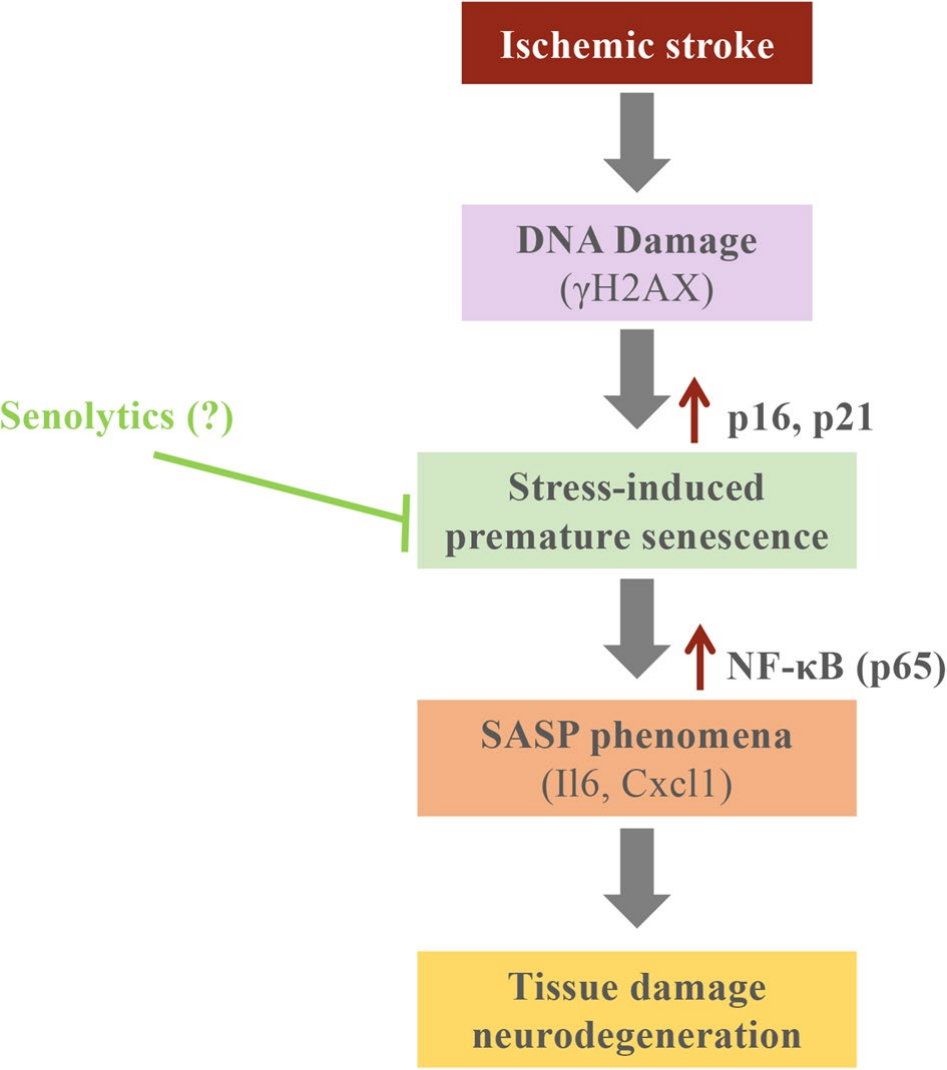
Graphical summary of the results.

In conclusion, there is an increase of senescence markers highly correlated with SASP cytokines restricted to the ischemic core in the tMCAO preclinical mouse model (Fig. 5). Furthermore, there is an increase of P16 positive cells surrounding the ischemic core in a human case of MCA stroke. These results would open new therapeutic opportunities for the emerging senolytic drugs, which selectively kill senescence cells. Further research would contribute to define the cellular phenotype of SASP in ischemic stroke and new therapeutic approaches would be determine for ischemic stroke patients.

## Supplementary Information

Supplementary Information.

## Data Availability

Requests for access to the data reported in this paper will be considered by the Lead contact.

## Abbreviations

SASP: Senescence-associated secretory phenotype
MCA: Middle cerebral artery
IL: Interleukin
tMCAO: Transient middle cerebral artery occlusion
rCBF: Regional cerebral blood flow
RT-qPCR: Real time quantitative polymerase chain reaction
CXCL1: Chemokine (C-X-C motif) ligand 1
CXCR2: Chemokine (C-X-C motif) receptor 2
NF-κB: Nuclear factor kappa-light-chain-enhancer of activated B cells
