# Supplementary material for: Acute ischemic stroke triggers a cellular senescence-associated secretory phenotype
Source: Sci Rep. 2021 Aug 3;11:15752. doi: 10.1038/s41598-021-95344-5 (PMC8333348; doi:10.1038/s41598-021-95344-5)

Supplemental Table S1 (sequence of RT-PCR primers)

Transcript name	Primer	Sequence
Actb	Forward	GTGACGTTGACATCCGTAAAGA
	Reverse	GCCGGACTCATCGTACTCC
p16	Forward	CCCAACGCCCCGAACT
	Reverse	GCAGAAGAGCTGCTACGTGAA
p21	Forward	TTGCCAGCAGAATAAAAGGTG
	Reverse	TTTGCTCCTGTGCGGAAC
Il6	Forward	ACCAGAGGAAATTTTCAATAGGC
	Reverse	TGATGCACTTGCAGAAAACA
Tnf-α	Forward	CAGGCGGTGCCTATGTCTC
	Reverse	CGATCACCCCGAAGTTCAGTAG
Cxcl1	Forward	ACTGCACCCAAACCGAAGTC
	Reverse	TGGGGACACCTTTTAGCATCTT
Tgf ß	Forward	ATAAAATCGACATGCCGTCC
	Reverse	TTGTTGAGACATCAAAGCGG
Cdk4	Forward	CAATGTTGTACGGCTGATGG
	Reverse	GGAGGTGCTTTGTCCAGGTA
Cxcr2	Forward	GAAATTTCGCCATGGACTTCTC
	Reverse	ACGAGCTAACAAAAGAAGGCCT

Figure S1. Morphological *p16* and *p21* expression characterization in a preclinical mouse model of stroke. Mosaic-stitched imatges of the infarct zone (16 to 18 imatges for each mosaic); white dots límits the infarcted area. Scale bar mosaic imatges = 500 µm. Scale bar zoom = 50 µm. Immunofluorescence controls, performed by omitting the primary antibodies, resulted in the abolition of the immunostaining in all cases (data not shown).

Figure S2. SA-B-gal expression in mouse brain tissue. Senescence-associated β-galactosidase (SA-β-gal) activity was not detected in tMCAO mice tissue. Scale bar = 100 µm.

Figure S3. Human sample globus pallidus ischemic stroke. *P16* and *P21* subcellular expression in globus pallidus ischemic stroke patient. Scale bar left images = 1000 µm (p16) and 500 µm (p21); middle images = 500 µm (p16) and 200 µm (p21); right images = 500 µm (p16), 200 µm (p16, p21).

Supplementary Figure S1

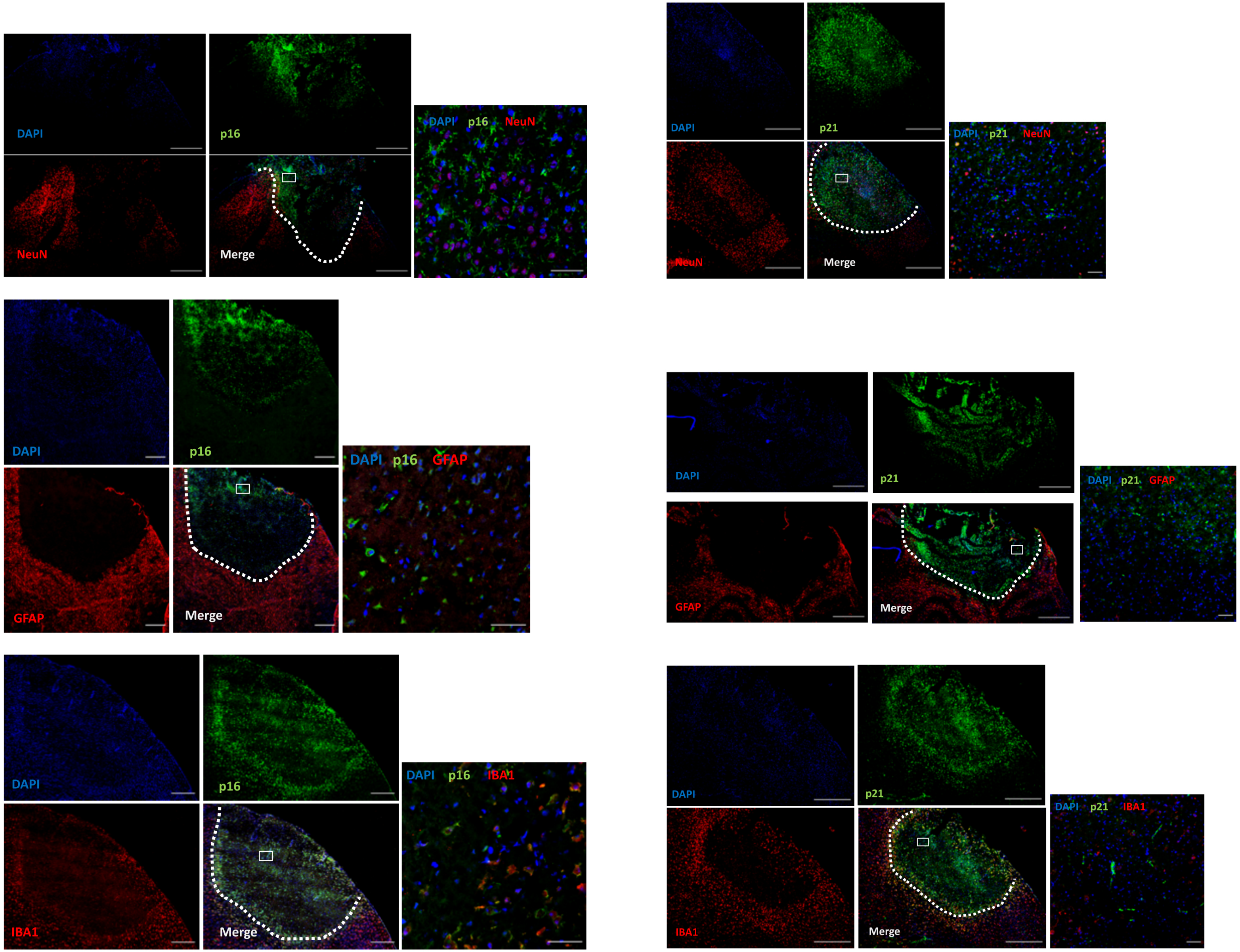

Supplementary Figure S2

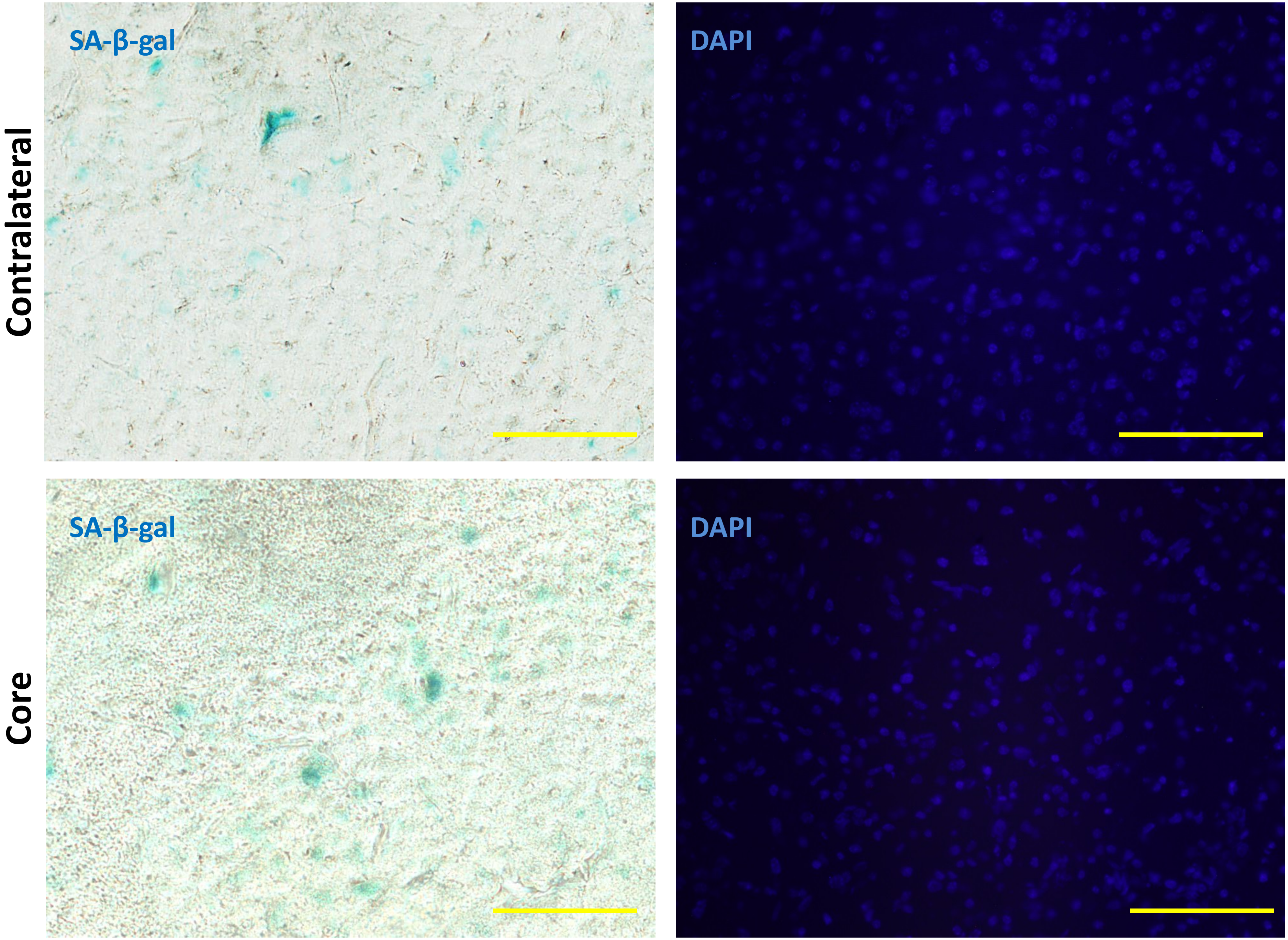

Supplementary Figure S3

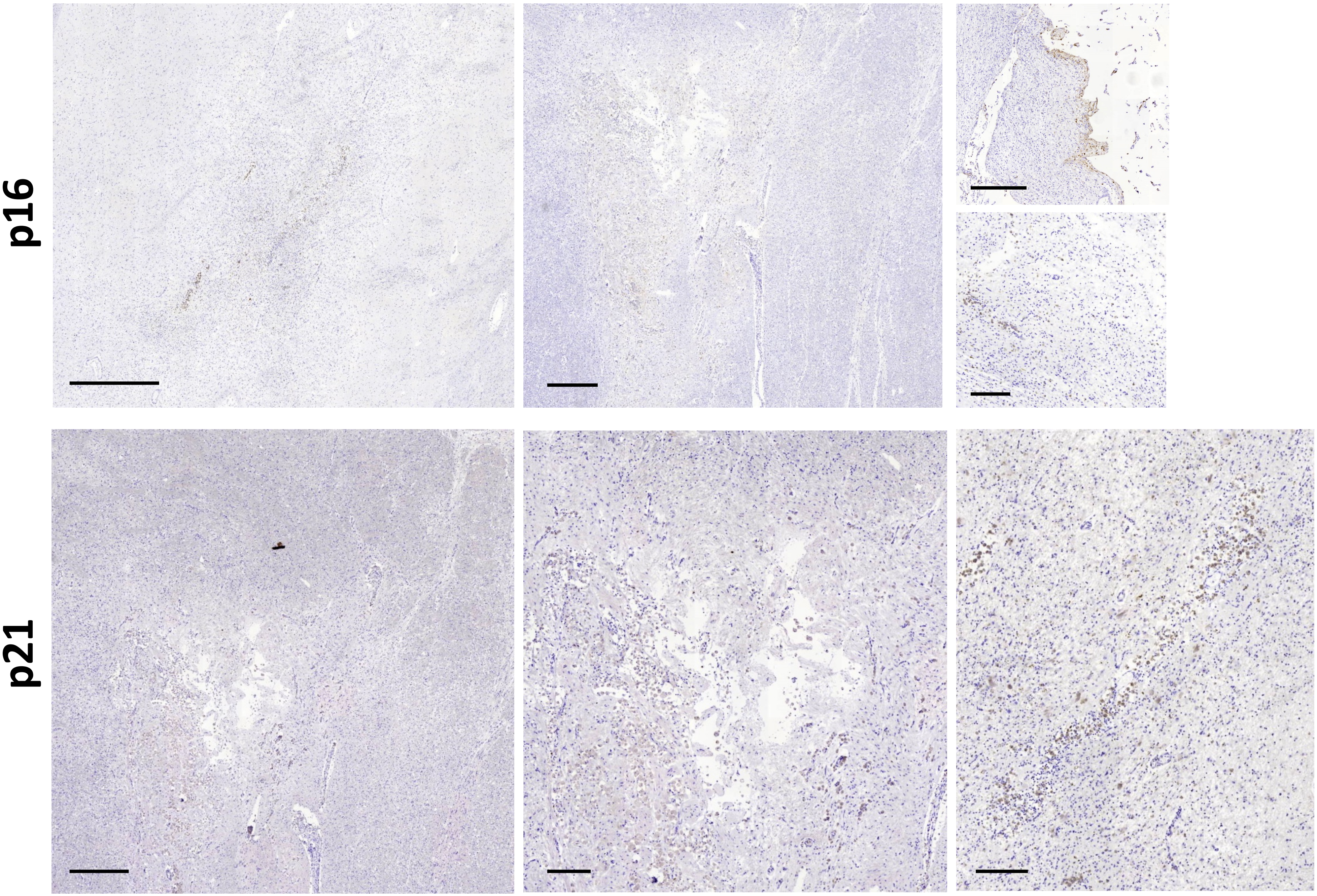

Supplement: Supplementary file 1 — Supplementary Information. [file 41598_2021_95344_MOESM1_ESM.pdf]
